# Anti-inflammatory α-Melanocyte-Stimulating Hormone Protects Retina After Ischemia/Reperfusion Injury in Type I Diabetes

**DOI:** 10.3389/fnins.2022.799739

**Published:** 2022-02-25

**Authors:** Rajesh Kumar Goit, Andrew W. Taylor, Amy C. Y. Lo

**Affiliations:** ^1^Department of Ophthalmology, Li Ka Shing Faculty of Medicine, The University of Hong Kong, Pokfulam, Hong Kong SAR, China; ^2^Department of Ophthalmology, Boston University School of Medicine, Boston, MA, United States

**Keywords:** retina, ischemia, reperfusion, diabetes, inflammation, oxidative stress

## Abstract

Retinal ischemia/reperfusion (I/R) injury is a major cause of vision loss in many ocular diseases. Retinal I/R injury is common in diabetic retinopathy, which as a result of hyperglycemia damages the retina and can cause blindness if left untreated. Inflammation is a major contributing factor in the pathogenesis of I/R injury. α-Melanocyte-stimulating hormone (α-MSH) is an anti-inflammatory peptide hormone that has displayed protective effects against I/R-induced organ damages. Here, we aimed to investigate the protective role of α-MSH on I/R-induced diabetic retinal damage using hyperglycemic C57BL/6J Ins2^Akita/+^ mice. Experimental I/R injury was induced by blocking the right middle cerebral artery (MCA) for 2 h followed by 2 h or 22 h of reperfusion using the intraluminal method. Since ophthalmic artery originates proximal to the origin of the MCA, the filament also blocked blood supply to the retina. Upon treatment with α-MSH at 1 h after ischemia and 1 h after reperfusion, animals displayed significant improvement in amplitudes of b-wave and oscillatory potentials during electroretinography. α-MSH also prevented I/R-induced histological alterations and inhibited the development of retinal swelling. Loss of retinal ganglion cells as well as oxidative stress were significantly attenuated in the α-MSH-treated retinae. Level of interleukin 10 was significantly increased after α-MSH treatment. Moreover, gene expression of glutamate aspartate transporter 1, monocarboxylate transporter (MCT) 1 and MCT-2 were significantly higher after α-MSH administration. In conclusion, α-MSH mitigates the severity of I/R-induced retinal damage under hyperglycemic condition. These beneficial effects of α-MSH may have important therapeutic implications against retinal I/R injury under hyperglycemic condition.

## Introduction

Retinal ischemia is a major cause of vision loss and blindness, and is a common feature in various retinal disorders such as glaucoma, diabetic retinopathy (DR), central retinal artery (CRA), or vein occlusion, carotid artery disease, blood hyperviscosity, and retinopathy of prematurity (Osborne et al., 2004). Retinal ischemia leads to depletion of oxygen and glucose, and in turn causes glutamate excitotoxicity, increase in intracellular calcium and disruption of blood-retinal barrier (BRB). However, the degree of retinal damage during ischemia depends on the severity and duration of the obstruction to blood flow (D’Onofrio and Koeberle, 2013). Although immediate reperfusion could attenuate the retinal damage, it results in leukocyte infiltration, inflammation and production of reactive oxygen species (ROS) that alter the retinal structure and function.

DR is a common microvascular complication of diabetes, and remains the leading cause of vision loss and blindness in the working adult population (Wang and Lo, 2018). In diabetes, hyperglycemia leads to biochemical and cellular changes that trigger production of ROS as well as inflammatory cytokines and chemokines. These mediators are major pathogenic factors causing retinal neuronal dysfunction, BRB breakdown and vascular leakage in early DR (Wang and Lo, 2018). Also, pericytes that line retinal capillaries are damaged due to sustained increase in PKCδ level, which are not reversed with return of normoglycemia (Geraldes et al., 2009). As a result, these changes can lead to localized areas of ischemia, edema, and angiogenesis, all of which can impair vision (Caldwell et al., 2003) through hemorrhage and tractional retinal detachment (Cai and Boulton, 2002). One of the current treatment options for DR is targeting vascular endothelial growth factor (VEGF), an angiogenic factor that is upregulated. However, some patients do not respond to anti-VEGF therapy (Antonetti et al., 2021). Also, anti-VEGF mediators can cause systemic complications due to their ability to enter systemic circulation (Simo and Hernandez, 2008). This demonstrates the need to develop new therapeutic approaches.

α-Melanocyte-stimulating hormone (α-MSH) is a tridecapeptide derived from the precursor hormone pro-opiomelanocortin (POMC). Mainly synthesized in the hypothalamus and the brain stem as well as in the pituitary gland, POMC-derived peptides have a wide range of actions such as energy homeostasis, steroidogenesis, pigmentation, thermoregulation, and anti-inflammation. Several studies showed that α-MSH has protective effects against ischemia/reperfusion (I/R) organ damages such as brain (Huh et al., 1997; Huang and Tatro, 2002; Aronsson et al., 2006; Chen et al., 2008; Savos et al., 2011; Goit et al., 2021), eye (Varga et al., 2013), heart (Vecsernyes et al., 2003), kidney (Chiao et al., 1997; Gong et al., 2004), and gut (Hassoun et al., 2002; Zou et al., 2003). Indeed, α-MSH counteracts retinal degeneration during early diabetes, and it alleviates the pro-inflammatory microenvironment caused by hyperglycemia in diabetic retina (Cai et al., 2018). Since individuals with type 1 diabetes have an increased risk of DR (Fong et al., 2004; Jansson et al., 2018), this study aimed to investigate the protective role of α-MSH on I/R-induced retinal damage upon hyperglycemia using type 1 diabetic mouse.

## Materials and Methods

### Animal

Twelve to fifteen weeks old C57BL/6J Ins2^Akita/+^ (Ins2^Akita/+^) mice were used as a model of type 1 diabetes in the present study. Since disease progression in female Ins2^Akita/+^ mice is slower and less uniform (Yoshioka et al., 1997) while estrogen exerts powerful neuroprotective actions during reproductive age following ischemic stroke in female mice (Brann et al., 2007; Suzuki et al., 2009), only male mice were included in this study. They were kept under standard laboratory conditions with 12 h light-dark cycle at the Centre for Comparative Medicine Research at The University of Hong Kong. All animal experiments followed the Cap. 340 Animals (Control of Experiments) Ordinance and Regulations and were approved by the Committee on the Use of Live Animals in Teaching and Research (CULATR 4837-18) at The University of Hong Kong.

One day before the experiment, blood glucose level was measured by Contour XT blood glucose meter (ELITE XL, Bayer HealthCare) and test strip (ELITE, Bayer HealthCare). Only mice with high blood glucose level (>20 mM) were included and randomly assigned to various experimental conditions. They were challenged with either sham operation or I/R injury with (i) 2 h of ischemia and 2 h of reperfusion or (ii) 2 h of ischemia and 22 h of reperfusion.

### Animal Model of Retinal Ischemia and Reperfusion

Retinal I/R injury was achieved by blocking blood flow into the middle cerebral artery (MCA) as previously described (Lo et al., 2005, 2007; Li et al., 2009, 2011, 2012; Goit et al., 2021). Briefly, animals were anesthetized with 2% isoflurane in 70% N_2_O and 30% O_2_ for induction and 1–1.5% isoflurane in 70% N_2_O and 30% O_2_ for maintenance during surgery. MCA occlusion (MCAO) was induced on the right side with a monofilament suture (Johnson and Johnson, Belgium) coated with vinyl polysiloxane impression material (3 M Dental Products, United States). The suture was introduced into the external carotid artery (ECA) and moved forward into the internal carotid artery (ICA) until the tip of the filament occluded the opening of MCA. The blockage was considered successful when a drop of more than 70% of baseline signal measured by the laser Doppler flowmeter (PeriFlux System 5000, Sweden) was observed. Since the origin of the ophthalmic artery (OA) is proximal to the origin of the MCA (Smith et al., 2002), the filament also blocked blood supply to the retina. Once the blockage was confirmed, the animals were transferred to an intensive care unit where they recovered from anesthesia. They were re-anesthetized for the removal of the filament and thus allowing reperfusion. The sham-operated animals underwent MCA exposure without occlusion. The core temperature during the surgery was maintained approximately at 37°C using a heating pad as described (Goit et al., 2021).

### α-Melanocyte-Stimulating Hormone Treatment

Animals were injected with 2, 10, 20, 50, 70, or 100 μg/mouse of α-MSH (Bachem AG, Switzerland) in phosphate-buffer saline (PBS) intraperitoneally at 1 h after ischemia and 1 h after reperfusion. Since α-MSH at 50 μg/mouse showed neuroprotective effects in cerebral I/R injury (Goit et al., 2021), this dosage was later used to further evaluate its protective effects in the I/R induced retinal damage. PBS was injected as a vehicle.

### Electroretinography

Mice were dark-adapted before electroretinography (ERG). For general anesthesia, the animal was injected intraperitoneally with a mixture of Ketamine and Xylazine (100 and 10 mg/kg body weight, respectively) in PBS. The pupil was locally anesthetized with 0.5% proparacaine hydrochloride (Alcon, Belgium) and dilated with 1% Mydriacyl (Alcon, Belgium). Gold wire electrodes (Diagnosys, United States) were placed on the apex of the cornea. A reference electrode (Chalgren, United States) was attached subcutaneously on the forehead and a ground electrode (Chalgren, United States) was placed subcutaneously at the tail region. Tears naturale II (Alcon, Belgium) was used to lubricate the eye during the recording. White flash of 3 (p)cd.s/m^2^ and 10 (p)cd.s/m^2^ intensities were delivered from the ColorDome Ganzfeld System (Diagnosys, United States). a-wave, b-wave, and the ascending phase of the b-wave (oscillatory potentials, OPs) were recorded using the Espion ERG Diagnosys and analyzed by Epsion V5 System (Diagnosys software) as previously reported (Li et al., 2012; Yang et al., 2017; Wang et al., 2020).

### Tissue Collection

After ERG recording, the animal was sacrificed by intraperitoneal injection of pentobarbital. Right eyeball was immediately enucleated and fixed with 4% paraformaldehyde (PFA) overnight at 4°C for histological and immunohistochemical staining. For Western blot and quantitative polymerase chain reaction (qPCR) techniques, the retina was snap frozen in liquid nitrogen immediately after collection.

### Histological Evaluation

4% PFA fixed eyeball was first dehydrated with graded series of ethanol and then immersed in chloroform; and finally embedded in paraffin. 5 μm thick sections were obtained using a microtome (Microm HM 315R, Germany). After deparaffinization and rehydration, sections containing the optic nerve head were stained with hematoxylin and eosin. Subsequently, sections were dehydrated and mounted with Permount™ (Thermo Fisher Scientific, United States). Image of the whole retinal section was taken under 20× magnification with a microscope (Eclipse 80i; Nikon, Japan) equipped with a digital camera (Diagnostic Instruments, Inc., United States) using Spot Advanced software (SPOT Imaging Solutions, United States). ImageJ was used to count the total number of retinal ganglion cell (RGC) in the ganglion cell layer (GCL) as well as to measure thickness of GCL, inner plexiform layer (IPL), inner nuclear layer (INL), outer plexiform layer (OPL), outer nuclear layer (ONL), and the whole retina. The central (150 μm from the optic nerve head) and the mid-peripheral (middle area between the optic nerve and the most peripheral part of the retina) regions of the retina were selected for thickness measurement. Cells with pyknotic nuclei in the GCL were excluded in the counting. Pyknotic cells were identified by the presence of condensed and darkly stained or sometimes fragmented nuclei (Perry et al., 1983).

### Immunohistochemistry

After deparaffinization and rehydration of sections containing the optic nerve head, antigen retrieval was achieved by incubation with proteinase K. Sections were blocked with 2% normal goat serum followed by overnight incubation with primary antibodies against glial fibrillary acidic protein (GFAP 1:500; Cat# ZO33429-2, Dako, Denmark), protein kinase C alpha (PKCα 1:1,000; Cat# sc-208, Santa Cruz Biotechnology, United States), calretinin (1:1,000; Cat# sc-11644, Santa Cruz Biotechnology, United States), and glutamine synthetase (GS 1:1,000; Cat# MAB302, Millipore, Germany). Subsequently, sections were incubated with the corresponding secondary antibodies (1:500; Molecular Probes, Invitrogen Corporation, United States) followed by incubation with 4′,6-diamidino-2-phenylindole (DAPI) and mounted for macroscopic analysis.

For DAB (3,3′-Diaminobenzidine) staining, 3% hydrogen peroxide was used to block endogenous peroxidase activity in sections after deparaffinization and rehydration. 10 mM sodium citrate was then used for antigen retrieval. Sections were blocked with 2% normal goat serum followed by overnight incubation with primary antibodies against poly-ADP-ribose (PAR 1:200; Cat# BML-SA216-0100, Enzo Life Sciences, United States) and nitrotyrosine (NT 1:200; Cat # 05-233, Merck KGaA, Germany). Subsequently, sections were incubated with the corresponding biotinylated secondary antibody (Vector Laboratories, United States) and avidin-biotin peroxidase complex (Vector Laboratories, United States). The immunoreactivity was developed with stable DAB (Invitrogen, United States) to generate the dark-brown color. After counterstaining with hematoxylin, sections were dehydrated and mounted for macroscopic analysis.

The immunohistochemical staining for each target antigen was performed in a single round including retinal sections from all experimental groups. The sections were then randomly coded and examined in a blinded approach. Photomicrographs were captured under 20× magnification. They were analyzed and scored according to the immunoreactivity intensity and distribution ranging from score 1 (absent or weak expression) to score 5 (strong expression) as previously reported (Li et al., 2009, 2011, 2012; Fu et al., 2012, 2015, 2017; Yang et al., 2017).

### Western Blot

Retinal tissue lysates were obtained from snap frozen retina using the radio immunoprecipitation assay buffer. Total protein concentration of the sample was determined by bicinchoninic acid (BCA) protein assay (Thermo Fisher Scientific™ Pierce™ BCA Protein Assay Kit, Thermo Fisher, United States) using bovine serum albumin (Bio-Rad, United States) as a protein standard. Proteins were separated with SDS-PAGE and transferred to a polyvinylidene difluoride membrane (Merck KGaA, Germany). After blocking in 5% skim milk in TBST (Tris-buffered saline, 0.1% Tween 20), the membrane was incubated overnight at 4°C with primary antibodies against interleukin 10 (IL-10 1:1,000; Cat# sc-1783, Santa Cruz Biotechnology, United States) and B-cell lymphoma 2 (Bcl-2 1:1,000; Cat# 2876, Cell Signaling Technology, United States). Glyceraldehyde 3-phosphate dehydrogenase (GAPDH 1:1,000; Cat# sc-32233, Santa Cruz Biotechnology, United States) was used as an internal loading reference. After incubation with the corresponding horseradish peroxidase-labeled secondary antibody (Vector Laboratories, United States), protein bands on the membrane were detected by enhanced chemiluminescence using the ChemiDoc MP Imaging System (Bio-Rad Laboratories, Inc., United States). The intensities of bands were quantified with ImageJ.

### Quantitative Polymerase Chain Reaction

RNA from snap frozen retina was extracted using TRIzol reagent (Invitrogen, United States). The concentration and quality of RNA were measured using a spectrophotometer (Hitachi Photodiode Array Bio-spectrophotometer U-0080D, Japan). Subsequently, 1 μg of total RNA was used as a template for cDNA synthesis using QunatiNova Reverse Transcription Kit (QIAGEN, Germany). cDNA equivalent to 50 ng of total RNA was subjected to qPCR using TB Green Master Mix (TaKaRa, Japan). The following sets of primers were used to determine the expression of glutamate and lactate related transporters in the retina: glutamate aspartate transporter (GLAST) 1 (Forward Primer 5′-GAATGGCGGCCCTAGATA GTA-3′; Reverse Primer 5′-CTTTCCGGGGTGGATGATGA-3′), monocarboxylate transporter (MCT) 1 (Forward Primer 5′-GGCTCCACTTAATCAGGCTTT-3′; Reverse Primer 5′-CAGGCCCTATTGGTCTCATCA-3′) and MCT-2 (Forward Primer 5′-GGGCTGGGTCGTAGTCTGT-3′; Reverse Primer 5′-ATCCAAGCGATCTGACTGGAG-3′). Beta-actin (Forward Primer 5′-GGCTGTATTCCCCTCCATCG-3′; Reverse Primer 5′-CCAGTTGGTAACAATGCCATGT-3′) was used as an internal reference. Each sample was tested in triplicate. The relative difference was expressed as the x-fold change of α-MSH-treated samples over vehicle-treated samples using the 2^–ΔΔCT^ method.

### Statistical Analysis

IBM SPSS Statistics Version 26 was used for statistical analysis. One-way ANOVA followed by Dunnett test was used to compare b-wave amplitude in ERG measurement. One-way ANOVA followed by Tukey’s test was used to compare retinal layer thickness, RGC count and OPs. Immunoreactivity was compared using Mann-Whitney *U*-Test. Independent samples *t*-test was used to compare Western blot and qPCR results. The results are presented as mean ± standard deviation. *p* < 0.05 was considered statistically significant.

## Results

### α-Melanocyte-Stimulating Hormone Improved b-Wave Amplitude and Oscillatory Potentials After I/R Injury

**FIGURE 1:**
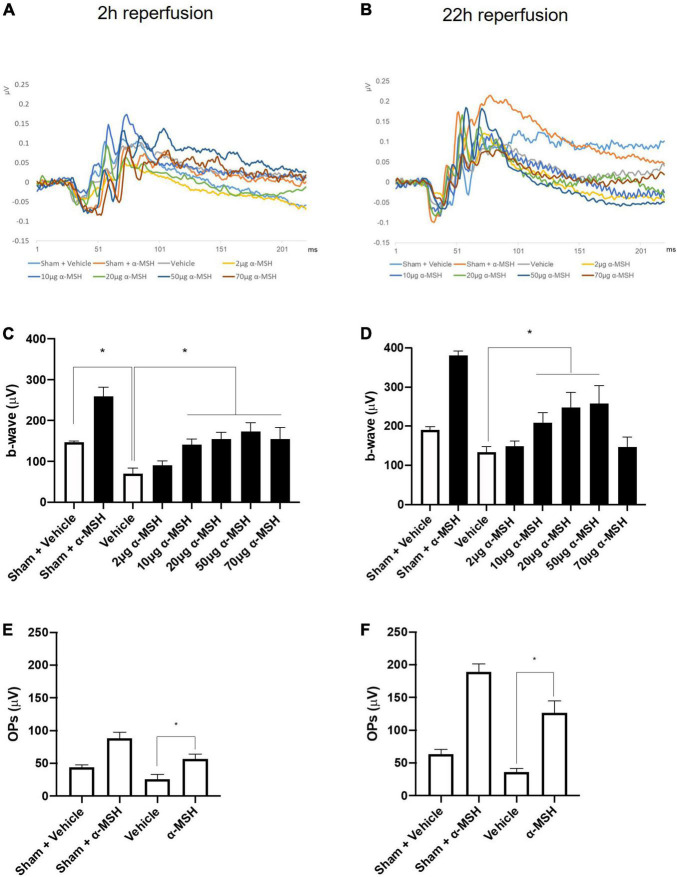
α-MSH preserved the retinal function after I/R injury. **(A)** Representative ERG waves at 2 h of reperfusion. **(B)** Representative ERG waves at 22 h of reperfusion. **(C)** The amplitude of b-wave was significantly higher in animals treated with 10, 20, 50, and 70 μg of α-MSH at 2 h of reperfusion. **(D)** This amplitude was significantly higher in animals treated with 10, 20, and 50 μg of α-MSH at 22 h of reperfusion. One-way ANOVA followed by Dunnett test. **(E,F)** OPs were significantly higher in α-MSH (50 μg) treated animals in both experimental conditions. One-way ANOVA followed by Tukey’s test. Flash 3 (P)cd.s/m^2^. *P < 0.05, *n* = 5–6 for each group.

The a-wave in ERG recording is associated with photoreceptors and the b-wave reflects the combined activity of depolarizing bipolar, amacrine, ganglion, and Müller cells (Kolb et al., 1995). In this study, the amplitude of a-wave was comparable between vehicle-treated animals and α-MSH-treated animals with/without retinal I/R injury (data not shown). However, retinal I/R injury resulted in significantly decreased b-wave amplitude in animals treated with vehicle when compared with sham-operated vehicle treated animals at 2 h of reperfusion. After retinal I/R injury, the amplitudes of b-wave were significantly higher in animals treated with 10, 20, 50, or 70 μg of α-MSH when compared with vehicle treatment (Figures 1A,C). At 22 h of reperfusion, a non-significant trend of decrease in b-wave amplitude was observed in vehicle-treated I/R injury animals when compared with vehicle-treated sham-operated animals (Figure 1D). More importantly, α-MSH treatment at 10, 20, or 50 μg significantly increased b-wave amplitudes when compared with vehicle treatment after I/R injury (Figures 1B,D). In addition, the b-wave amplitude in each experimental group appeared to be higher at 22 h of reperfusion when compared with that at 2 h of reperfusion (Figures 1A–D). In this study, the b-wave implicit times were also analyzed and compared; yet, no difference could be found among vehicle-treated and α-MSH-treated groups.

The OPs in ERG recording were calculated as the sum of the amplitudes of OP1, OP2, OP3, and OP4. After retinal I/R injury, they were significantly higher in animals treated with 50 μg of α-MSH compared with vehicle-treated animals at both 2 h and 22 h of reperfusion (Figures 1E,F). Animals treated with other doses of α-MSH did not yield significant improvements in OPs (data not shown).

### α-Melanocyte-Stimulating Hormone Preserved Retinal Structure After I/R Injury

**FIGURE 2:**
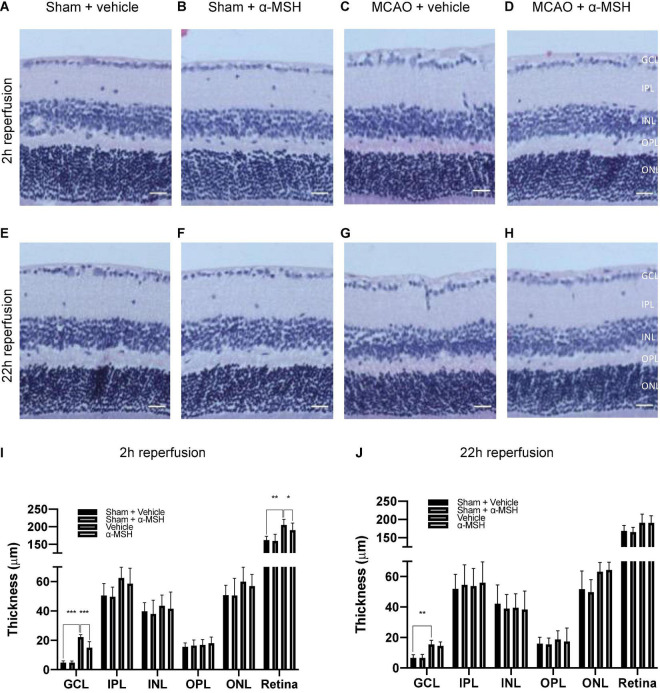
α-MSH preserved morphological integrity of retina after I/R injury. **(A–D,I)** Thicknesses of GCL and total retina were significantly higher in animals treated with vehicle compared with α-MSH-treated animals or vehicle-treated sham animals at 2 h of reperfusion. **(E–H,J)** Only thickness of GCL was significantly higher in vehicle-treated animals compared to vehicle-treated sham animals at 22 h of reperfusion. *P < 0.05, **P < 0.01, ***P < 0.001, One-way ANOVA followed by Tukey’s test, *n* = 6 for each group. Scale bar = 25 μm.

Fifty microgram/mouse of α-MSH had been shown to be neuroprotective in cerebral I/R injury (Goit et al., 2021). It also yielded beneficial effects in recovering b-wave amplitude and OPs in ERG measurements as describe above. Therefore, we further evaluated its protective effects in retinal damage at this dosage. Firstly, thickness of various retinal layers was measured in the central and the mid-peripheral parts of the retina from sham/MCAO-challenged animals with/without α-MSH treatment. In the central retina, thickness of individual layer was comparable among all experimental groups except that of GCL and total retinal at 2 h of reperfusion. The thickness of GCL and total retina was significantly higher in vehicle-treated I/R injury animals when compared with sham-operated vehicle control or I/R injury α-MSH treated animals (Figures 2A–D,I), suggesting the presence of retinal swelling after I/R injury in vehicle-treated animals. Thus, α-MSH treatment resulted in preservation of retinal morphology and protection from swelling after I/R injury. However, thicknesses of various retinal layers were comparable between groups after retinal I/R injury at 22 h of reperfusion (Figures 2E–H,J). Thickness measurement was also performed in the mid-peripheral retina but no significant differences could be observed at 2 h as well as 22 h of reperfusion (data not shown).

**FIGURE 3:**
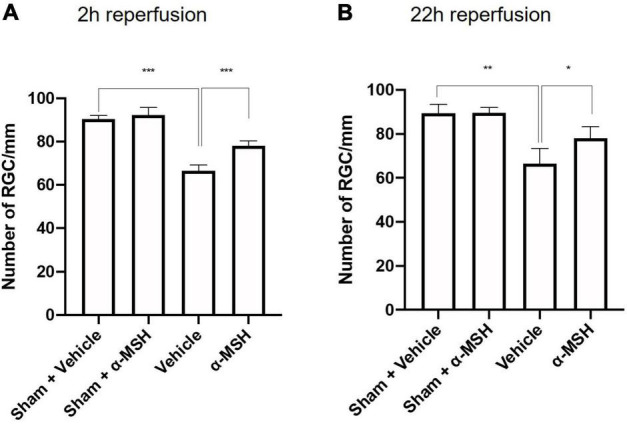
α-MSH maintained the number of RGC in the GCL. **(A,B)** Significantly lower number of RGC was recorded in vehicle-treated animals compared with α-MSH-treated animals or vehicle-treated sham animals in both experimental conditions. *P < 0.05, **P < 0.01, ***P < 0.001, One-way ANOVA followed by Tukey’s test, *n* = 6 for each group.

The number of viable RGC in GCL was counted. α-MSH treatment did not impose a change in RGC number with sham operation (Figures 3A,B). After I/R injury, the number of viable RGC was significantly decreased in vehicle-treated animals when compared with those in sham-operated animals at both 2 h and 22 h of reperfusion (Figures 3A,B). More importantly, α-MSH treatment significantly prevented decrease in the RGC count at both 2 h and 22 h of reperfusion, suggesting its ability in attenuating the damage upon retinal I/R injury.

### Immunohistochemistry Analysis

**FIGURE 4:**
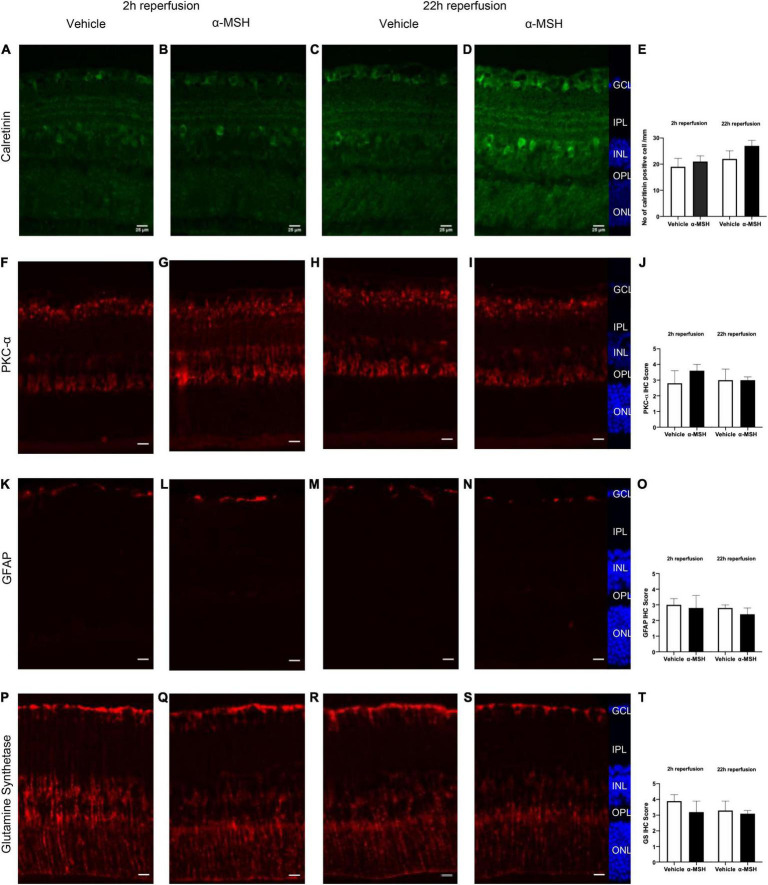
Immunoreactivities of retinal neuronal and glial cells after I/R injury. **(A–D)** Calretinin expression was higher in α-MSH-treated animals at 22 h of reperfusion. **(E)** Number of calretinin positive cell/mm. **(F–I)** PKC-α expression was higher in α-MSH-treated animals at 2 h of reperfusion. **(J)** IHC scores for PKC-α.**(K–N)** GFAP expression was similar between the groups in both experimental conditions. **(O)** IHC scores for GFAP. **(P–S)** α-MSH reduced the GS expression at 2 h of reperfusion. **(T)** IHC scores for GS. Mann-Whitney *U*-Test, *n* = 6 for each group. Scale bar = 25 μm.

The calcium binding protein calretinin is a known marker for amacrine cells in the retina (Fu et al., 2015). Normally, calretinin immunoreactivity can be observed in the INL and in three distinct strata in the IPL (Figures 4A–D). At 2 h of reperfusion, there was no difference in calretinin immunoreactivity between vehicle-treated and α-MSH-treated retinae (Figures 4A,B,E). However, higher calretinin expression was observed in α-MSH-treated animals at 22 h of reperfusion (Figures 4C–E). Meanwhile, PKC-α is abundantly expressed in retinal bipolar cells (Fu et al., 2015). There was a trend of increase in its expression in animals treated with α-MSH at 2 h of reperfusion (Figures 4F,G,J). However, this trend was not sustained at 22 h of reperfusion (Figures 4H–J).

Retinal glial activation was also assessed. GFAP, a well-known marker for astrocytes (Fu et al., 2015), was present mostly in the GCL. Its immunoreactivity was similar between vehicle-treated and α-MSH-treated groups in both experimental conditions (Figures 4K–O). Meanwhile, the expression of GS, a marker for Müller cells in the retina was also examined. At 2 h of reperfusion, GS expression appeared to be higher in vehicle-treated animals when compared with α-MSH-treated animals (Figures 4P,Q,T). GS level after vehicle treatment was decreased to a level similar to that in α-MSH-treated retinae at 22 h of reperfusion (Figures 4R–T).

**FIGURE 5:**
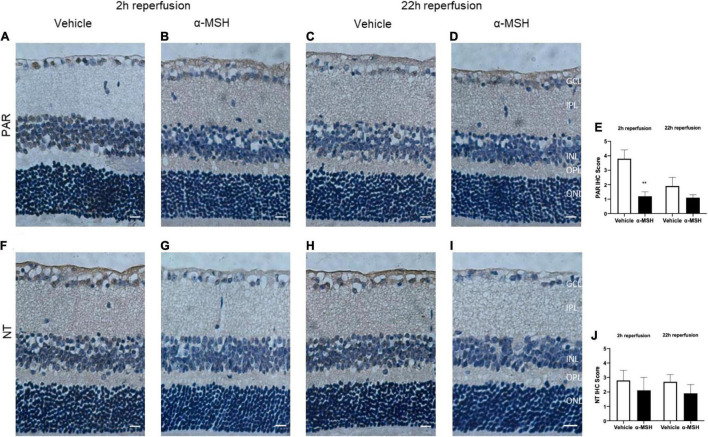
α-MSH decreased the oxidative and nitrative stress after I/R injury. **(A–D)** Significantly weaker PAR immunoreactivity was observed in animals treated with α-MSH at 2 h of reperfusion. This immunoreactivity was comparable at 22 h of reperfusion. **(E)** IHC scores for PAR. **(F–I)** Weaker NT immunoreactivity was observed in α-MSH-treated animals in both experimental conditions. **(J)** IHC scores for NT. **P < 0.01, Mann-Whitney *U*-Test, *n* = 6 for each group. Scale bar = 25 μm.

Immunoreactivity of PAR, an indicator of nitric oxide-dependent DNA damage, was localized in the GCL and INL and was significantly higher in vehicle-treated animals compared with α-MSH-treated animals at 2 h of reperfusion (Figures 5A,B,E). Thus, α-MSH treatment was able to significantly decrease PAR immunoreactivity at 2 h of reperfusion. At 22 h of reperfusion, PAR immunoreactivity was comparable (Figures 5C–E). Immunoreactivity of NT, a marker of reactive nitrogen species-induced nitrative stress, showed a non-significant trend of decrease in animals treated with α-MSH when compared with vehicle-treated animals at 2 h as well as 22 h of reperfusion (Figures 5F–J).

### α-Melanocyte-Stimulating Hormone Increased Expression of Anti-Inflammatory Protein After I/R Injury

**FIGURE 6:**
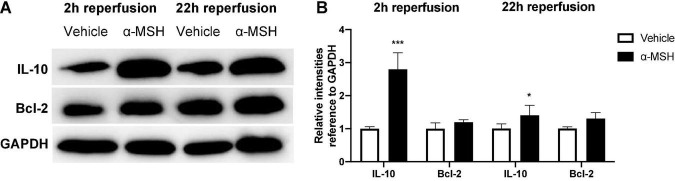
α-MSH increased expressions of anti-inflammatory and pro-survival proteins after I/R injury. **(A,B)** Protein expressions of IL-10 was significantly higher in animals treated with α-MSH at 2 h and 22 h of reperfusion. **(A,B)** Although Bcl-2 expression was higher in animals treated with α-MSH in both experimental conditions, the expression was comparable. *P < 0.05, ***P < 0.001, Independent samples *t*-test, *n* = 5–6 for each group.

The anti-inflammatory and anti-apoptotic properties of α-MSH was analyzed by Western blot. After I/R injury, IL-10 expression was significantly higher with α-MSH treatment at both 2 h and 22 h of reperfusion (Figures 6A,B). On the other hand, expression of Bcl-2 was unchanged with/without α-MSH treatment in both experimental conditions (Figures 6A,B).

### α-Melanocyte-Stimulating Hormone Increased Gene Expression of Glutamate and Lactate Transporters After I/R Injury

**FIGURE 7:**
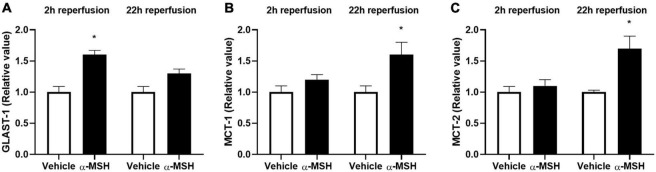
α-MSH increased expression of glutamate and lactate related genes after I/R injury. **(A)** GLAST-1 expression was significantly higher in animals treated with α-MSH at 2 h of reperfusion. **(B,C)** MCT-1 and MCT-2 expressions were significantly higher in animals treated with α-MSH at 22 h of reperfusion. **(A–C)** GLAST-1 expression at 22 h of reperfusion and expressions of MCT-1 and MCT-2 at 2 h of reperfusion were comparable. *P < 0.05, Independent samples *t*-test, *n* = 5 for each group.

Glutamate release is a common phenomenon during/after ischemia. Glutamate uptake into Müller cells is a major mechanism in regulating extracellular glutamate concentration in the retina (Rauen et al., 1998). Therefore, the gene expression of GLAST-1, a glutamate transporter in Müller cells, was determined by qPCR. GLAST-1 gene expression was significantly increased in animals treated with α-MSH at 2 h of reperfusion but not 22 h of reperfusion (Figure 7A). Meanwhile, gene expressions of lactate transporters, MCT-1 and MCT-2, were also assessed. MCT-1 and MCT-2 gene expressions were significantly increased in animals treated with α-MSH at 22 h of reperfusion but not 2 h of reperfusion (Figures 7B,C).

## Discussion

Retinal ischemia induces a wide range of deleterious effects, including excitotoxicity, inflammation, and oxidative stress. These events finally contribute to the death of retinal neurons and several vision-threatening diseases. Similarly, DR is associated with reduced retinal blood flow, dysfunction of the retinal neurons, followed by breakdown of BRB and finally changes in visual function.

In the eye, α-MSH suppresses proinflammatory signals, promotes production of anti-inflammatory cytokines, transiently lowers intraocular pressure, protects neuronal cells from damage, and modulates immune response (Lee et al., 2009; Taylor and Lee, 2010; Clemson et al., 2017). Anti-oxidative and anti-apoptotic effects of α-MSH was also reported when it was administrated intravitreally in early DR (Zhang et al., 2014). Besides, this neuropeptide plays a role in inhibiting BRB breakdown and vascular leakage as well as improving electrophysiological functions and morphology of retina in early DR (Cai et al., 2018). More importantly, it has no known adverse effects (Clemson et al., 2017) and is already under clinical trial for advanced dry age-related macular degeneration (ClinicalTrials.gov, 2018). A melanocortin agonist, PL9643 as ophthalmic solution for subjects with dry eye is progressing into late-stage clinical trials after improvements in dry eye disease in a phase 2 study (ClinicalTrials.gov, 2020; Palatin, 2021). In addition, melanocortin receptor agonists are under investigation for their therapeutic potential in inflammatory ocular diseases such as non-infectious uveitis, DR, and diabetic macular edema (Palatin, 2021). All these results suggest the beneficial effects of α-MSH in DR. The microvascular changes in the diabetic retina often result in ischemia, after which retinal neurons die over a long period of time. Therefore, there is a possible therapeutic window to rescue these neurons destined to die (Antonetti et al., 2021). So, the possible protective role of α-MSH on I/R-induced retinal damage in type 1 diabetic mouse was investigated in this study.

The a-wave is considerably less affected by ischemia than the b-wave, and b-wave amplitude has been used as a quantitative marker of reduced retinal blood flow as well as experimental ischemic retinal injury despite normal retinal histology (Osborne et al., 2004). Among α-MSH-treated animals, b-wave amplitude was reduced in animals treated with either lower (2 μg) or higher (70 μg) concentrations of α-MSH. This suggests that α-MSH follows a bell-shaped dose-response curve (Holdeman and Lipton, 1985; Goit et al., 2021). The significantly decreased b-wave amplitude in vehicle-treated animals in both experimental conditions may be related to the level of GS expression proportional to the extracellular glutamate concentration (Shen et al., 2004), a sign of retinal stress and pathology (Bringmann et al., 2009). In darkness, photoreceptors release glutamate to activate receptors on bipolar and horizontal cells, and rapid clearance plus recycling of glutamate is critical for effective neurotransmission. In this study, animals were dark adapted before ERG recording; decreased b-wave amplitude in the vehicle-treated animals could be linked to a transient functional impairment of Müller cells in response to ischemia resulting in insufficient recycling of glutamate. High level of glutamate in the synaptic zones between photoreceptor cells and ON-bipolar cells saturates the glutamate receptors on the bipolar cells in darkness. This leads to a decline in the amplitude of b-wave in light (Winkler et al., 1999). Therefore, one possibility would be the inability of α-MSH to raise extracellular glutamate concentration to a level high enough to induce higher expression of GS. This may explain why reduced GS expression was observed with α-MSH treatment despite the beneficial effect of GS. Similarly, OPs indicate amacrine cells activity in the inner retina (Wachtmeister and Dowling, 1978) and OPs amplitude loss is a significant parameter of retinal ischemia (Jehle et al., 2008). Moreover, low expression of GLAST-1, a glutamate transporter, in vehicle-treated animals indicate the possibility of a temporary functional impairment of Müller cells after I/R injury with inadequate recycling of glutamate. The high intravitreal glutamate levels after suppression of glutamate transporter leads to elevated ganglion cell death (Vorwerk et al., 2000) and might affect impulse conduction. Treatment with α-MSH increased the gene expression of GLAST-1 as well as increased immunoreactivities of PKC-α and calretinin, and in turn improved b-wave amplitude and OPs after I/R injury.

In sham operated animals, the amplitude of b-wave and OPs were higher in α-MSH treated animals compared to vehicle treated animals. It might be due to animals used in our study were dark adapted before ERG recording. More ATP is consumed in the dark due to Na^+^/K^+^ ATPase activity in the inner segment of rod photoreceptor. As lactate is produced, high photoreceptor metabolism may be maintained with lactate shuttles (Country, 2017). Lactate is taken up by Müller cells and oxidized to pyruvate to sustain oxidative metabolism (Lindsay et al., 2014). The lactate transporters, MCTs, are expressed in retinal cells and inhibition of MCTs causes reduction of b-wave and OPs (Bui et al., 2004). In addition, it has been suggested that hyperglycemia increases retinal metabolism and increases amplitudes of b-wave (Klemp et al., 2004) and OPs (Algvere and Gjotterberg, 1974) in diabetic patients without retinopathy compared to euglycemia. Both human and animal studies showed that α-MSH is involved in the management of glucose and slow progression of diabetes (Costa et al., 2006), suggesting its role in glucose metabolism. Interestingly, opposite effects have been observed in rodent studies. α-MSH when present in the central nervous system can increase insulin sensitivity but may contribute to insulin resistance when circulate in the periphery (Costa et al., 2006). At this point it is unclear which is the dominating effect in our investigations, although we cannot rule out the possibility that α-MSH’s effect on ERG may be mediated *via* its influence on glucose metabolism.

The retina has a dual blood supply to sustain high metabolic function; the outer layers being supplied by the choroid capillaries and the inner layers being supplied by the CRA. Therefore, the severe effects of ocular ischemia are less frequently observed due to collateral vessels and retrograde blood flow in the retina (Shuaib et al., 2011). As a result, the blood flow to the retina is reduced but not completely blocked after MCAO (Kaja et al., 2003) and b-wave recovers after reperfusion (Osborne et al., 2004). Significantly higher amplitude of b-wave in animals treated with α-MSH compared to vehicle treated animals in both experimental conditions supported that α-MSH decreases the recovery time after retinal I/R injury. In this study, amplitude of b-wave and OPs were higher in 22 h of reperfusion groups compared to 2 h of reperfusion groups. This difference was due to short and long dark adaptation times. As the dark adaptation time increases, the amplitude of ERG waves increases gradually (Yu et al., 2007).

Inward movement of sodium, calcium and chloride ions along with water after ischemia can cause cell swelling and cytotoxic edema (Lipton, 1999). After retinal I/R injury, marked swelling of the ganglion cell bodies and their processes precede loss of these cells (Stevens et al., 2002). Indeed, there was an increase in the thickness of GCL and total retina in animals treated with vehicle after I/R injury, in agreement with the presence of retinal edema. Importantly, this increase in retinal thickness was ameliorated by α-MSH treatment, which supports the protective effects of α-MSH in preventing cell swelling and thus helps to maintain retinal structural integrity after I/R injury.

It has been shown that antioxidants play a role in the recovery of b-wave amplitude, preservation of retinal morphology and rescue of neurons in GCL (Li et al., 2009, 2011; Wang et al., 2020). Stronger PAR immunoreactivity in GCL and INL might be another possible reason for delayed recovery of b-wave amplitude and histological alternations in vehicle-treated animals after I/R injury. Despite the decreased amplitude of b-wave and number of damaged ganglion cells in vehicle-treated animals, we did not detect TUNEL-positive cells in retinal sections after I/R injury (data not shown). This agreed with our observation that Bcl-2 expression was comparable with/without α-MSH treatment in both experimental conditions. Bcl-2 is an anti-apoptotic protein which expression is important for retinal survival after ischemia (Martinou et al., 1994; Park et al., 2013). This suggests that ischemic damage may also occur by non-apoptotic mechanisms (Steele et al., 2008; Allen et al., 2014). It might also be due to the considerably longer survival of ischemic retina than the ischemic brain due to 100-time higher concentration of neuroglobin, a neuron-specific respiratory protein, in the mouse retina than in the mouse brain (Schmidt et al., 2003). Moreover, glucose concentration is higher in the retina when compared with the brain (Tang et al., 2000). This higher glucose concentration might likely fuel the retina during ischemia. In addition, vitreous humor bathes the retina and oxygen in the vitreous body makes it possible for the retina to tolerate ischemia (Hayreh and Weingeist, 1980). Besides, we only recorded laser-Doppler reading from the MCA but not from the OA. There is a possibility that the brain and the retina received different levels of occlusion. In addition, previous studies indicate that the amount of cell death is related to the method used to induce retinal ischemia. For example, occlusion of retinal vessels induces less severe consequence than increase of the intraocular pressure above systemic blood pressure (Hayreh and Weingeist, 1980).

Moreover, α-MSH has a role in anti-inflammation because retinal expression of IL-10 was upregulated in animals treated with α-MSH after I/R injury. IL-10 suppresses inflammation and angiogenesis in most ocular inflammations (Ghasemi et al., 2012). It modulates the expression of cytokines and chemokines that medicate inflammation as well as attenuates endothelial dysfunction and vasoconstriction mediated by oxidative stress after ischemia (Garcia et al., 2017). Importantly, higher IL-10 level correlates with its protective function against DR (Mysliwiec et al., 2006; Lee et al., 2008).

It has been shown that pre-existing hyperglycemic conditions including diabetes provide substantial structural and functional protection to the ischemic retina (Osborne et al., 2004). However, hyperglycemia-induced oxidative stress and inflammation are primary pathogenic factors for DR. Also, degenerative changes in the ganglion cell and nerve fiber layers are prominent pathological features of DR. The reduction of oxidative stress and inflammatory effect are among the most important therapeutic strategies to slow down DR (Wang et al., 2020). In this study, α-MSH downregulated ROS production and upregulated expression of anti-inflammatory protein in addition to maintaining structural and functional integrities after I/R injury. Since neurodegenerative retinal disease such as DR alters expression of MCTs, these transporters may be important therapeutic targets (Kolko et al., 2016). Increased expressions of MCT-1 and MCT-2 in α-MSH-treated animals suggest a role for this neuropeptide in retinal metabolism through lactate signaling and transport. Thus, these beneficial effects of α-MSH may have important therapeutic implication against retinal I/R injury under hyperglycemic condition. As the present study was limited by inclusion of Ins2^Akita/+^ mice only, further studies using non-diabetic mice are required to confirm whether α-MSH only acts on the retina or it also normalizes systemic changes due to hyperglycemia in order to restore normal structure and function of the retina.

## Data Availability Statement

The original contributions presented in the study are included in the article/supplementary material, further inquiries can be directed to the corresponding author/s.

## Ethics Statement

The animals were handled according to the conditions of the Cap. 340 Animals (Control of Experiments) Ordinance and Regulations, and all relevant legislation and Codes of Practice in Hong Kong. All the animal handling and experimental methods were approved by the Committee on the Use of Live Animals in Teaching and Research (CULATR 4837-18) at The University of Hong Kong.

## Author Contributions

AL designed the idea and supervised the study. RG performed the experiments, analyzed the data, and wrote the manuscript. AL and AT revised the manuscript and approved the final manuscript. All authors contributed to the article and approved the submitted version.

## Conflict of Interest

The authors declare that the research was conducted in the absence of any commercial or financial relationships that could be construed as a potential conflict of interest.

## Publisher’s Note

All claims expressed in this article are solely those of the authors and do not necessarily represent those of their affiliated organizations, or those of the publisher, the editors and the reviewers. Any product that may be evaluated in this article, or claim that may be made by its manufacturer, is not guaranteed or endorsed by the publisher.
